# Effect of Hydrothermal Degradation on Flexural Fatigue Strength of Various Cubic-Containing Translucent Zirconia

**DOI:** 10.1055/s-0042-1755630

**Published:** 2022-10-11

**Authors:** Santiphab Kengtanyakich, Chaimongkon Peampring

**Affiliations:** 1 Department of Prosthetic Dentistry, Faculty of Dentistry, Prince of Songkla University, Songkhla, Thailand

**Keywords:** zirconia ceramic, translucent zirconia, cubic phase, flexural fatigue strength, hydrothermal degradation

## Abstract

**Objective** The aim of this study is to investigate the fatigue and hydrothermal degradation behavior among the cubic-containing translucent yttrium oxide stabilized tetragonal zirconia polycrystal (Y-TZP).

**Materials and Methods** Four groups of commercial Y-TZP (T, ST, XT, and P), containing different amount of cubic crystal, were examined. Artificial aging was accomplished by autoclaving at 122°C under 2 bar pressure for 8 hours. Fatigue simulation parameters were set using an axial 50 N load, a total of 240,000 cycles. Flexural fatigue strength was evaluated.

**Statistical Analysis** Two-way analysis of variance with Tukey's honestly significant difference test was used to determine the difference in fatigue strength values between various type of zirconia materials within control and aging with a significant level of 5%. Weibull analysis was conducted on the fatigue strength data.

**Results** All groups showed the mean flexural fatigue strength had no significant difference in hydrothermally aged specimens compared with specimens without aging ( *p* = 0.154). Group T showed significantly higher flexural fatigue strength followed by group ST, XT, and P. Group XT and P showed no significant difference in strength value ( *p* > 0.05). Group T demonstrated a lower probability of failure than group ST, XT, and P whether with or without aging. Weibull modulus in group T and ST with aging condition was increased and higher than all the experimental groups.

**Conclusion** Cubic-containing zirconia materials (group ST, XT, and P) exhibited lower flexural fatigue strength than non-cubic 3Y-TZP zirconia (group T). However, various cubic-containing translucent zirconia was assumed to have high resistance to hydrothermal degradation.

## Introduction

Yttrium-stabilized tetragonal zirconia polycrystal (Y-TZP) is widely used in dentistry because of its excellent strength and fracture toughness after machining and sintering compared with other dental ceramics. 1 Y-TZP has been stabilized with addition of yttria; as a result, tetragonal crystal structure can be retained at room temperature. Tetragonal zirconia is metastable and can return to a more stable monoclinic phase when it is initiated by stress and/or humid environments. 2 The volume expansion associated with the tetragonal to monoclinic phase transformation (t→m transformation) results in compressive stress; therefore, crack propagation in the ceramic can be terminated. 1 It is considered as a high performance ceramic which is clinically indicated for multiple purposes.

The t→m transformation can also spontaneously occur over time at low temperature. When the t→m transformation is not triggered by the local stress, this phenomenon is known as aging, hydrothermal degradation, or low-temperature degradation (LTD). 3 Although some studies showed that low temperature aging did not promote any deleterious impact on the mechanical properties of Y-TZP ceramic. 4 On the contrary, many studies indicated that the drawbacks of LTD would be the deterioration of zirconia's mechanical properties. 5 6 7 The fatigue resistance is one of the main concerning properties of ceramic materials because it affects the serviceability of Y-TZP restorations in the patient's mouth. Crack propagation under intermittent loading could reproduce the clinical condition and predict the mechanical behavior of ceramics. 8

The increased patients' esthetic demands for both anterior and posterior teeth are driving the development of highly translucent monolithic zirconia. Translucency is considered as one of the most important factors in controlling esthetics and plays an important role in material selection. 9 Translucent zirconia materials can be modified by several factors: grain size, pores, pressure applied, and material thickness. 10 11 Moreover, increasing of Y _2_ O _3_ stabilizer tends to alter the phase structure from tetragonal to cubic phase resulting in increased translucency and improved hydrothermal aging resistance. 12 13 The amount of cubic crystal structures seems to have an effect on the microstructure of zirconia materials. 13 Nowadays, the studies have reported that increasing amount of cubic phase in zirconia can improve translucency of cubic-containing zirconia compared with conventional zirconia. 13 14

However, the clinical recommendations for using translucent zirconia restorations cannot bring clear advantage and there is little information on literature regarding the properties especially in fatigue resistance and the degradation behavior of recently introduced translucent zirconia for monolithic restorations, particularly the cubic-containing zirconia with the varying amount of cubic structure. Therefore, the aim of this study was to investigate the fatigue resistance and hydrothermal degradation behavior among commercial translucent zirconia products containing different amount of cubic crystal (< 30%, 30 to 50%, and > 50% cubic phase) and compare them with conventional zirconia (0% cubic phase). The null hypothesis was that no difference in flexural fatigue strength between hydrothermally aged and nonhydrothermally aged of various cubic-containing translucent zirconia.

## Materials and Methods

Four different commercially available zirconia materials were tested in this study ( Table 1 ). Twenty zirconia disk-shaped specimens for each group were fabricated by a computer-aided designing/computer-aided machining system. A milling machine (Coritec 250i, IMES-ICORE, Eiterfeld, Germany) was used to mill disk-shaped specimens out of the zirconia blanks. All zirconia specimens were fully sintered with a high temperature furnace (VITA Zyrcomat 6000 MS, Vita Zahnfabrik, Bad Säckingen, Germany) following the respective manufacturer's firing protocols ( Table 2 ). The final specimen dimensions for each specimen were confirmed with digital vernier caliper (Mitutoyo, Mitutoyo Manufacturing Company Ltd, Kawasaki, Japan); 15 mm diameter, 1 mm thick. Afterwards, all specimens were ultrasonically cleaned (Model: 460/M, Elma Schmidbauer GmbH, Singen, Germany) in distilled water for 10 minutes. Ten specimens were aged hydrothermally using an autoclave (TOMY ES 215/ ES-315, Tomy Kogyo Co. Ltd., Nerima, Japan) at 122°C under 2-bar pressure over a period of 8 hours ( *n* = 10/group) and other 10 specimens were kept in the air dry at room temperature ( *n* = 10/group) before being subjected to cyclic loading.

**Table 1:** Summary of materials used in the present study

Material	Type	Code	Main composition a (% by weight)	Amount of cubic structure a (% by volume)	Lot number	Manufactures
Vita YZ T	3Y-TZP	T	ZrO _2_ 90–95% Y _2_ O _3_ 4–6% Al _2_ O _3_ 0–1%	0	77970	VITA Zahnfabrik, Bad Säckingen, Germany
Vita YZ ST	4Y-TZP	ST	ZrO _2_ 88–93% Y _2_ O _3_ 6–8% Al _2_ O _3_ 0–1%	< 30	56730	VITA Zahnfabrik, Bad Säckingen, Germany
Vita YZ XT	5Y-TZP	XT	ZrO _2_ 86–91% Y _2_ O _3_ 8–10% Al _2_ O _3_ 0–1%	30–50	61960	VITA Zahnfabrik, Bad Säckingen, Germany
Prettau Anterior	4–6Y-TZP	P	ZrO _2_ main component Y _2_ O _3 _ < 12% Al _2_ O _3 _ < 1%	> 50	ZB6124B	Zirkonzahn GmbH, Bruneck, Italy

a Manufacturer's data.

**Table 2:** Sintering protocol of each group

Group	Heating rate (°C/min)	Final temperature (°C)	Holding time (min)	Cooling protocol
T	17	1530	120	Cooling off at 100% to 200°C
ST	8	1530	120	Cooling off at 100% to 200°C
XT	4	1450	120	Cooling off at 100% to 200°C
P	8	1500	120	Cooling rate 8°C/min to 50°C

All specimens were cyclic loaded in a chewing simulator (CS 4.4, SD Mechatronic GmbH, Feldkirchen-Westerham, Germany) using an axial load of 50 N for 240,000 cycles with a frequency of 1.1 Hz. A stainless steel metal stylus (Ø = 2 mm) was used as an antagonist abrader and the force was transferred to the middle site of the specimen by opposing metal stylus under water at room temperature. After cyclic loading was completed, specimens were subjected to a biaxial flexural strength test according to ISO 6872. 15 Disk-shaped specimens were positioned on three support balls (Ø = 2.5 mm), which were placed 11 mm apart from each other in a triangular position. A flat circular tungsten piston (Ø = 1.6 mm) was used to apply an increasing load (0.5 mm/min) until catastrophic failure using a universal testing machine (Lloyd instruments, Model LRX-Plus, AMETEK Lloyd Instrument Ltd., Hamphshire, United Kingdom) with 5 kN load cell. Flexural fatigue strength was calculated according to this following equation:

*σ* = [–0.2387 *P* ( *X* – *Y* )]/ *b* ^2^

where *σ* is the maximum tensile stress (MPa), *P* is the total load to fracture ( *N* ), *b* is the thickness at fracture origin (mm), and *X* and *Y* are calculated according to:

*X* = (1 + *ν* ) ln( *r*_2_ / *r*_3_ ) ^2^ + [(1– *ν* )/2)]( *r*_2_ / *r*_3_ ) ^2^

*Y* = (1 + ν)[1 + ln( *r*_1_ / *r*_3_ ) ^2^ ] + (1–ν)( *r*_1_ / *r*_3_ ) ^2^

where *ν* is Poisson's ratio ( *ν* = 0.30), 8 *r*_1_ is the radius of the support circle (6.05 mm), *r*_2_ is the radius of the loaded area (0.8 mm), and *r*_3_ is the radius of the specimen (7.5 mm). The flexural fatigue strength testing was performed on both specimens with and without hydrothermal aging and compared statistically.

The fracture patterns were used in the descriptive fractographic analysis of fractured specimens using scanning electron microscopy (SEM; JSM-5800 LV [SEM5800], JEOL, Japan) at ×200 and ×500 magnification.

### Statistical Analysis

Mean flexural fatigue strength and standard deviations were recorded. Assessment of normality distribution and homogeneity of variance were performed using the Shapiro–Wilk test and Levene's test, respectively. The data was normally distributed and homogeneity of variance was equal in each group. Therefore, two-way analysis of variance with Tukey's honestly significant difference test was used to determine the difference in flexural fatigue strength values between various type of zirconia materials in with LTD aging and without LTD aging groups ( *n* = 10/group). Weibull analysis was conducted on the fatigue strength data for evaluating strength reliability. The description of the Weibull distribution is given by 16 :

*P*_f_ = 1–exp[–( *σ* / *σ*_0_ ) ^m^ ]

where *P*_f_ is the probability of failure, *σ* is the fatigue strength at a given *P*_f_ , *σ*_0_ is the characteristic strength (MPa), and *m* is the shape parameter (Weibull modulus). The statistical analysis was done using SPSS software, version 24.0 (IBM Corporation, Armonk, New York, United States) to detect statistically significant differences ( *α* = 0.05).

## Results

The statistical analysis showed a significant difference in flexural fatigue strength among the material groups ( *p* < 0.05), while LTD showed no significant effect on strength degradation in all groups ( *p* = 0.154). The mean flexural fatigue strength showed significantly highest value in group T (815 MPa without aging group and 811 MPa with aging group). Group ST was significantly higher than group XT and P in specimens with and without aging. However, group XT and P showed no significant difference in flexural fatigue strength value regardless of aging condition ( *p* > 0.05) ( Table 3 ). Moreover, all data showed no statistically significant interaction between material type and aging factor ( *p* = 0.184).

**Table 3:** Mean ± SD of flexural fatigue strength (MPa)

Group	Without LTD aging	With LTD aging
T	815.47 ± 43.44 ^Aa^	811.45 ± 27.8 ^Aa^
ST	683.39 ± 29.34 ^Ba^	652.63 ± 22.56 ^Ba^
XT	431.51 ± 20.48 ^Ca^	365.06 ± 19.67 ^Ca^
P	406.36 ± 23.83 ^Ca^	334.88 ± 23.41 ^Ca^

Abbreviations: LTD, low-temperature degradation; SD, standard deviation.

Note: Different uppercase letters in the same column indicate statistically significant difference ( *p* < 0.05); different lowercase letters in the same row indicate statistically significant difference ( *p* < 0.05).

**Fig. 1:**
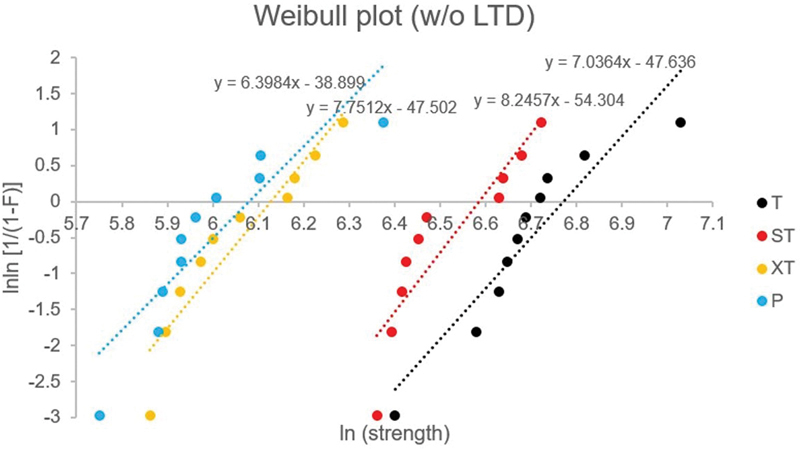
Weibull graph for all experimental groups without aging condition. Weibull parameter estimates are shown for comparison.

**Fig. 2:**
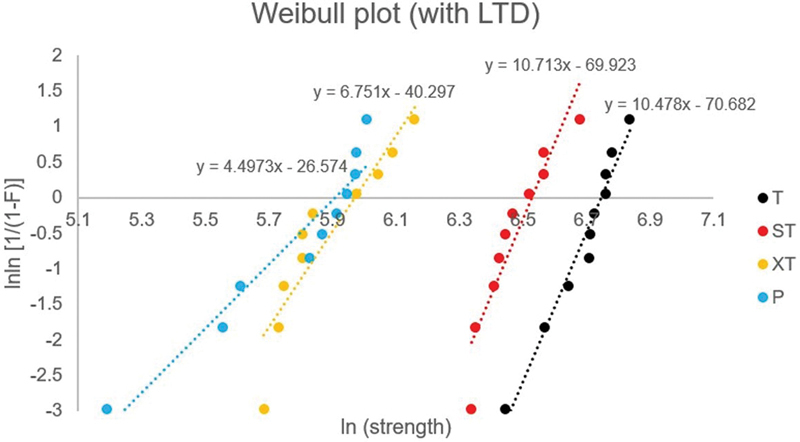
Weibull graph for all experimental groups with aging condition. Weibull parameter estimates are shown for comparison.

Weibull parameters including Weibull modulus ( *m* ) and characteristic strength ( *σ*_0_ ) are listed in Table 4 . Group T demonstrated a lower probability of failure and a higher strength than group ST, XT, and P whether with or without LTD aging. With LTD aging condition, the Weibull modulus in group T and ST were increased and higher than all experimental groups. The characteristic strength ( *σ*_0_ ) was higher for group T than that for group ST, XT, and P, respectively, in both of aging conditions. The probability of failure distribution curve is shown in Figs. 1 and 2 . Group ST, XT, and P demonstrated a shift to the left of the distribution curve in both of aging conditions.

**Table 4:** Weibull parameters of each experimental group; Weibull modulus ( *m* ) and characteristic strength ( *σ*_0_ )

Group	Without LTD aging		With LTD aging	
	*m*	*σ*_0_ (MPa)	*m*	*σ*_0_ (MPa)
T	7.04	871.22	10.48	850.48
ST	8.25	724.66	10.71	683.35
XT	7.75	458.66	6.75	391.11
P	6.40	436.81	4.50	368.34

Abbreviation: LTD, low-temperature degradation.

**Fig. 3:**
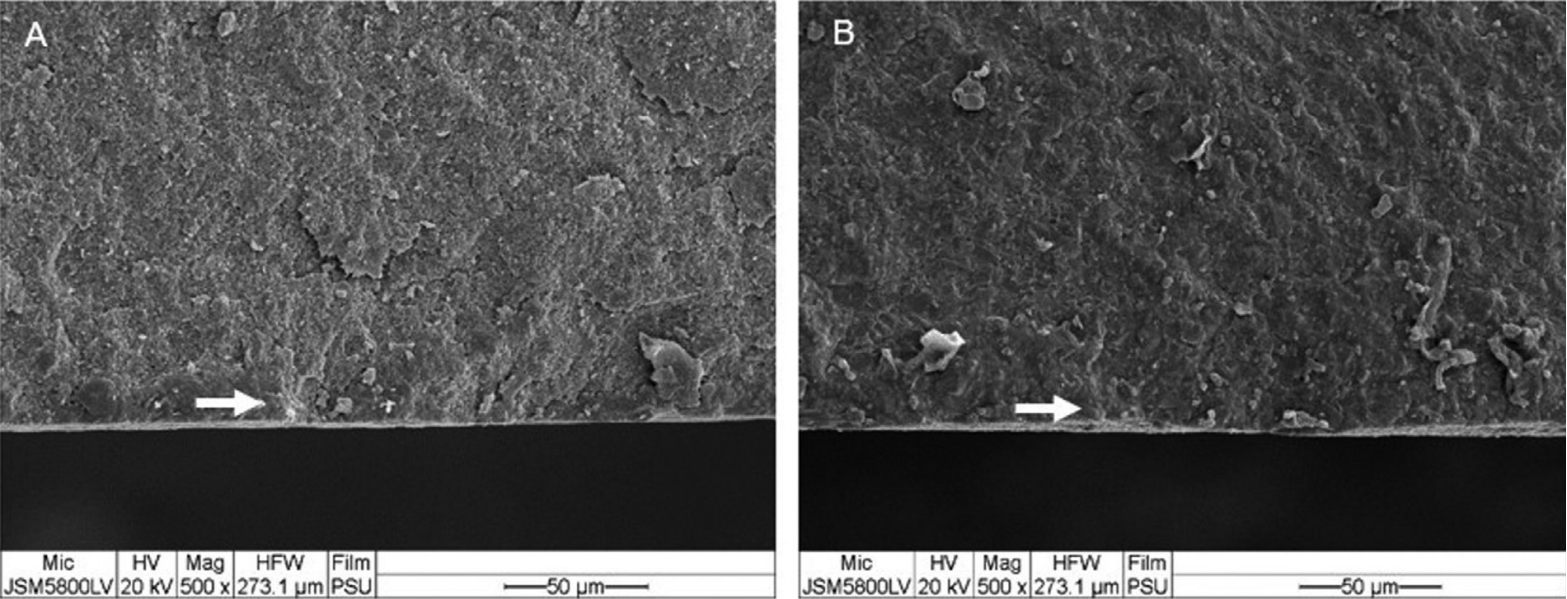
Fracture surface of ( **A** ) group T (non-cubic) and ( **B** ) group P (cubic) after flexural testing. Tension surface of specimen is facing down and the white arrows indicated the crack origin (magnification ×500).

**Fig. 4:**
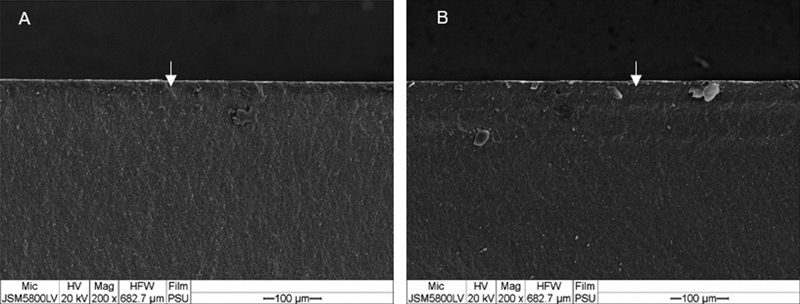
Fracture surface of nonaged groups. ( **A** ) Group T (non-cubic) and ( **B** ) group P (cubic) after flexural testing show twist hackle at top (compression) surface. The crack was running in the direction of the white arrows (magnification ×200).

**Fig. 5:**
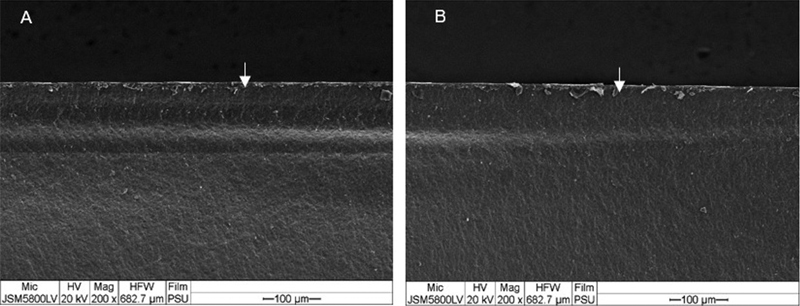
Fracture surface of hydrothermally aged groups. ( **A** ) Group T (non-cubic) and ( **B** ) group P (cubic) after flexural testing show twist hackle at top (compression) surface. The crack was running in the direction of the white arrows (magnification ×200).

SEM analysis showed that all experimental groups created the same fracture surface patterns. The crack lines could be traced back to an origin of fracture at tension surface of specimen ( Fig. 3 ). Hydrothermally aged and nonaged groups showed twist hackle and a crack propagating from top to bottom running perpendicular to the initial stress field as shown in Figs. 4 and 5 .

## Discussion

This recent study used various commercial products, which could be classified based upon the amount of cubic structure according to manufacturers' guideline as low (< 30% by volume), moderate (30–50% by volume), and high cubic-containing zirconia (> 50% by volume). From the result of this study, there was a statistically significant difference in flexural fatigue strength among the material groups, therefore the null hypothesis was partially rejected.

The fatigue resistance is one of the main concerning properties of ceramic materials because it affects the life of Y-TZP restorations. Crack propagation under intermittent loading could lead to catastrophic failure of restorations intraorally. 8 *In vitro* cyclic loading condition under humid environment simulates the clinical function of the restoration. The fatigue simulation parameters in this study were set using an axial 50 N load, a total of 240,000 cycles according to previous study. 17 Zirconia containing cubic crystal (group ST, XT, and P) showed significant decrease in flexural fatigue strength. It was because the cubic structure is the most stable phase which eliminates the transformation after receiving an applied stress reducing the potential for stress-induced transformation toughening. 18 The lack of this mechanism resulted in decreased fatigue strength of these materials. Moreover, Zhang et al in 2016 found that zirconia with largest grain size had the lowest strength. 19 In our previous study, the effect of different cubic-containing translucent zirconia on microstructure characteristics was evaluated. It was found that the average grain size for group XT was 1.57 µm which was larger than the group T and ST. Group P had the largest grain sizes of 2.28 µm. 7 Therefore, larger grain sizes of group XT and P might contribute to lower mechanical strength compared with smaller grains of group T and ST due to the dislocation on the crystal grain boundaries under dynamic or fatigue assessment affecting the transposition of the stimuli from grain to grain. 19 20

The strength in present results was higher than a previous study which evaluated the flexural fatigue strength of 3Y-TZP, 4Y-PSZ, and 5Y-PSZ on a 3-point bend test for 6,000 cycles. It was reported that the flexural fatigue strength of 3Y-TZP, 4Y-PSZ, and 5Y-PSZ were 640, 304, and 232 MPa, respectively. 21 It could be explained that different testing methods and material thickness might affect differences in results. The flaws of ceramic specimen for 3-point bend test were increased at the edge of each side and it could easily propagate in undesirable edge failures. Therefore, the flexural strength decreased. 22 The thickness of specimen could influence mechanical properties of the material. In this study, the thickness of specimen was set at 1 mm because it is considered as an ideal thickness for the fabrication of monolithic zirconia restorations. 23 However, the result in this study showed the same trend as previous study which was declination of fatigue strength of cubic-containing zirconia.

Autoclave aging was used to simulate the LTD aging condition. This study investigated *in vitro* accelerated hydrothermal aging characteristics of material using autoclave condition at 122°C under 2-bar pressure over a period of 8 hours. At this temperature, the nucleation was detected by small surface upheavals which corresponded to the transformation of one or a few grains. Consequently, this condition was used to detect the tetragonal to monoclinic phase transformation. It is possible to assume that 1 hour of autoclave aging in this condition has the same effect as 1 year in clinical services. 2 24 This study found that fatigue strength were not reduced significantly after hydrothermal aging. It might be explained that the flexural fatigue strength is the bulk property, in which, the effect site of hydrothermal aging was only at the surface and did not penetrate to the internal core of the material. Although the hydrothermal degradation or LTD can also spontaneously t→m transform over time in the presence of water or water vapor at relatively low temperatures, some studies reported that monoclinic phase was detected and affected the surface properties, however, the bulk properties did not alter after aging. 5 25

Weibull distribution was performed in the present study because the strength properties of ceramic materials are controlled by the number of surface flaws and flaws size distribution. Therefore, it is possible to provide reliability data relating to the fracture of ceramic materials. Weibull parameters including Weibull modulus or shape parameter ( *m* ) and characteristic strength ( *σ*_0_ ) estimate failure probability expression. 16 A lower Weibull modulus indicates more variation in flaws distribution in the material. On the other hand, a large Weibull modulus indicates the flaw sizes are all similar in size resulting in greater structural reliability. In the present study, all groups without LTD aging revealed negligible difference in Weibull modulus. In LTD aging groups, group T and ST showed higher Weibull modulus than other groups. It might be explained that LTD is a process in which the transformation of tetragonal to monoclinic phase occurs because of humidity. Formation of the hydroxyl ions (OH ^-^ ) in water molecules can diffuse into the zirconia lattice then water reacts with ZrO _2_ or Y _2_ O _3_ bonds at crack tips. As a consequence, Zr-OH or Y-OH bonds are formed and the Zr-OH or Y-OH bond brings about the lattice strain on the surface. Strain accumulation creates nucleation sites for phase transformation on the surface; as a result, the metastable tetragonal transformation to stable monoclinic phase can occur with a large volume expansion. 26 27 For this reason, the surface flaws or microcracks increased from this phenomenon and flaws were all similarly distributed compared with the group without LTD aging in which microcracks occurred randomly in some areas of the surface. When fracture load was applied to the specimen, increasing of microcracks under loading was found. Group T and ST possessed high amount of metastable tetragonal structure and were prone to t→m transformation from previous mechanism. Therefore, group T and ST had higher Weibull modulus and hence had greater structural reliability after aging. Group XT and P that contained high amount of cubic crystal caused a more stable condition of zirconia; therefore, t→m transformation did not occur. 18

The Weibull characteristic strength ( *σ*_0_ ) is the strength at a probability of failure of 63.2% for a test specimen and loading protocol. From the current study, the Weibull characteristic strength values were coinciding with the mean flexural fatigue strength. Group ST, XT, and P demonstrated a shift to the left of the distribution curve and indicated a decrease in flexural fatigue strength, in both of aging conditions. Group T presented high Weibull characteristic strength because of the higher amount of tetragonal structure; therefore, transformation toughening phenomenon could take place. As a result, strength for group T was greater than the other groups. 28 29

The fractographic analysis showed that all the fractures initiated at the initial stress of load application site and crack propagated to the side of tensile stress. Twist hackles were formed when the stress field had been changed and they were generated during the crack propagation. 30 31 Tension surface of the fractured discs showed crack origin which were mainly semi-elliptical flaws and surrounded with hackle lines. When twist hackles were formed and a crack propagating from top to bottom ran perpendicular to the initial stress field, catastrophic fracture of specimen took place. The pattern of grain fracture was not evaluated in this study; however, transgranular crack is a common pattern of crack propagation in zirconia. 32 Further studies are required to investigate the properties of the cubic-containing commercial translucent zirconia with long-term aging. Furthermore, the *in vivo* studies and wear characteristic of the cubic-containing commercial translucent zirconia should be evaluated.

## Conclusion

Based on the limitations of this *in vitro* study, it was concluded that cubic-containing zirconia materials (group ST, XT, and P) exhibited lower flexural fatigue strength than non-cubic 3Y-TZP zirconia (group T). However, hydrothermal aging in autoclave for 8 hours did not cause a statistically significant decrease in the flexural fatigue strength of all experimental groups.
